# Pharmacological myeloperoxidase (MPO) inhibition in an obese/hypertensive mouse model attenuates obesity and liver damage, but not cardiac remodeling

**DOI:** 10.1038/s41598-019-55263-y

**Published:** 2019-12-10

**Authors:** Arnold Piek, Debby P. Y. Koonen, Elisabeth-Maria Schouten, Eva L. Lindtstedt, Erik Michaëlsson, Rudolf A. de Boer, Herman H. W. Silljé

**Affiliations:** 1 Department of Cardiology, University Medical Center Groningen, University of Groningen, Groningen, The Netherlands; 2 Department of Pediatrics, Section Molecular Genetics, University Medical Center Groningen, University of Groningen, Groningen, The Netherlands; 3 Early Cardiovascular, Renal and Metabolism, R&D BioPharmaceuticals, AstraZeneca, Gothenburg, Sweden

**Keywords:** Risk factors, Cardiology, Drug development, Experimental models of disease

## Abstract

Lifestyle factors are important drivers of chronic diseases, including cardiovascular syndromes, with low grade inflammation as a central player. Attenuating myeloperoxidase (MPO) activity, an inflammatory enzyme associated with obesity, hypertension and heart failure, could have protective effects on multiple organs. Herein, the effects of the novel oral available MPO inhibitor AZM198 were studied in an obese/hypertensive mouse model which displays a cardiac phenotype. Eight week old male C57BL6/J mice received 16 weeks of high fat diet (HFD) combined with angiotensin II (AngII) infusion during the last 4 weeks, with low fat diet and saline infusion as control. Treated animals showed therapeutic AZM198 levels (2.1 µM), corresponding to 95% MPO inhibition. AZM198 reduced elevated circulating MPO levels in HFD/AngII mice to normal values. Independent of food intake, bodyweight increase and fat accumulation were attenuated by AZM198, alongside with reduced visceral adipose tissue (VAT) inflammation and attenuated severity of nonalcoholic steatohepatitis. The HFD/AngII perturbation caused impaired cardiac relaxation and contraction, and increased cardiac hypertrophy and fibrosis. AZM198 treatment did, however, not improve these cardiac parameters. Thus, AZM198 had positive effects on the main lipid controlling tissues in the body, namely adipose tissue and liver. This did, however, not directly result in improved cardiac function.

## Introduction

Unhealthy lifestyle is a strong driver of obesity, diabetes mellitus (DM) and hypertension. Currently, in Western countries more than 30% of the adult population is obese, 28% has hypertension and more than 8% has DM^1–3^. It is expected that these prevalences will even further increase in the coming years. Moreover, obesity, diabetes and hypertension are important risk factors for cardiovascular diseases^4^ and, therefore, modifying these risk factors or their systemic effects could be an interesting preventive or therapeutic approach.

Obesity and diabetes affect tissue metabolism, but are also associated with numerous systemic changes that may impact on other organs including the heart. Amongst others, obesity results in a hyperdynamic circulation with increased blood volume, while inducing (neuro)hormonal changes and promoting atherosclerosis and myocardial ischemia^5^. Both obesity and diabetes also adversely affect vascular function and are closely linked to hypertension^6–9^. Hypertension on its turn has direct hemodynamic effects, requiring increased cardiac workload, thereby promoting heart failure development^10^. Importantly, these risk factors also induce vascular- and systemic-inflammatory states and elevated plasma levels of inflammatory factors are observed in subjects with these risk factors^9,11–14^. Systemic and endothelial inflammation has been postulated to play an important role in the development of heart failure with preserved ejection fraction (HFpEF) (ejection fraction, EF, >50%)^9,11–16^. In contrast to heart failure with reduced ejection fraction (HFrEF) (EF, <40%), this syndrome is poorly understood and no-proven therapy exists. The exact characteristics are still under debate, but increased cardiac wall thickness and reduced diastolic function (relaxation) with preserved EF (>50%) are some of the main features^4^. Inflammation appears to be an overarching phenomenon with all these conditions and targeting inflammatory factors may therefore be beneficial at multiple organ and tissue levels in obese, diabetic, hypertensive and HFpEF patients.

The inflammatory enzyme myeloperoxidase (MPO), which is mainly produced by neutrophils and can be detected in the circulation, is a possible therapeutic target. The major role of MPO is in host defense, but it is also associated with obesity, insulin resistance, hypertension and heart failure^17–22^. In heart failure patients, plasma MPO levels are elevated and associated with worse cardiac function, and have been reported to be a predictor of heart failure outcome^23–25^. Moreover, network analysis suggested a prominent role for MPO in the HFpEF network^20^. MPO is also associated with the development of non-alcoholic steatohepatitis (NASH) and MPO plasma levels and hepatic MPO activity have been reported to increase in patients with NASH^26–28^. A causal relationship has been inferred from mouse studies showing that mice that have no MPO (MPO^−/−^ mice) on a high fat diet (HFD) showed less adipose inflammation and fat accumulation^29^. Furthermore, mouse studies have provided evidence that MPO can contribute to cardiac remodelling and also myocardial fibrosis, and MPO deficiency could preserve cardiac function, and reduce atrial fibrillation and ventricular tachycardia under pro-fibrotic conditions, present in severe heart failure models, like in post-myocardial infarction^30–33^.

Altogether, MPO presents an attractive target for pharmacological interference in obesity and hypertension, with possible (in)direct cardiac protective effects. In this study, we investigated the effects of a novel orally available MPO inhibitor, AZM198, which belongs to the class of 2-thioxanthines^34^*. In vitro* and *in vivo* studies have shown high specificity towards MPO at low micromolar plasma levels^34,35^. AZM198 effects on adipose tissue, liver and the heart were investigated in a combined obesity and hypertension mouse model with cardiac HFpEF characteristics.

## Results

### The obesity associated increase in MPO plasma levels was attenuated by AZM198 treatment

**Figure 1:**
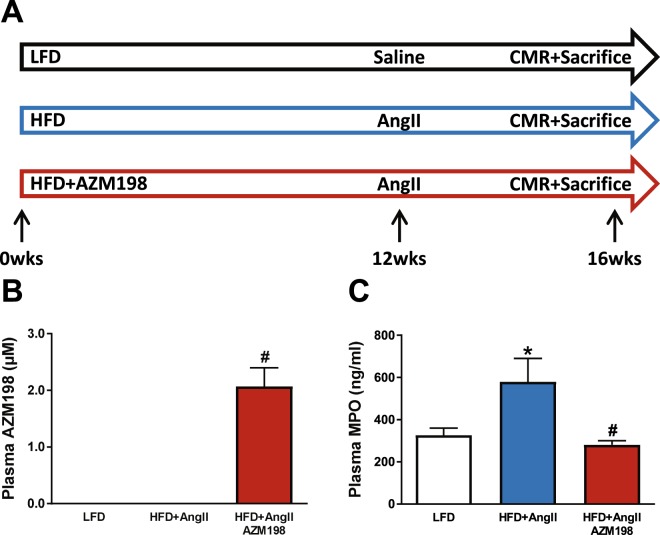
Experimental design and blood plasma levels of AZM198 and MPO. (**A**) Schematic experimental overview of the obesity/hypertension mouse model. Three experimental groups were included. Mice received either high fat diet (HFD) or low fat diet (LFD) for 16 weeks. HFD was supplemented with MPO-inhibitor (AZM198) or no inhibitor. During the last 4 weeks, infusion of angiotensin II (AngII) or saline was performed using osmotic minipumps. Prior to sacrifice, cardiac magnetic resonance imaging (CMR), body composition analysis and intraperitoneal glucose tolerance tests (IPGTT) were performed. Mice were sacrificed at 16 weeks. (**B**) Blood plasma levels of AZM198 at sacrifice. (**C**) Blood plasma levels of myeloperoxidase (MPO) at sacrifice. N = 11–12. Bars represent means. Error bars represent standard error of the mean. *P < 0.05 as compared to LFD. ^#^P < 0.05 as compared to HFD + AngII.

Mice were provided a control (LFD) diet or a high fat diet (HFD) with or without AZM198 for 16 weeks. During the last 4 weeks, hypertension was induced in the obese animal groups by infusion of AngII (HFD/AngII groups) (Fig. 1A). Food intake measurements showed no differences in food intake between diet supplemented with AZM198 and the respective placebo HFD diet (Supplemental Table 2). At the end of the experiment, plasma AZM198 levels were determined and this revealed that AZM198 could be detected in all treated mice with a mean level of 2.1 ± 0.3 µM (Fig. 1B). This experimental setting did not allow for measuring specific myeloperoxidase (MPO) activity in blood plasma. Therefore, sufficient exposure to the drug in the HFD/AngII group was concluded by comparing the data with systemic AZM198 exposures in parallel zymosan-induced peritonitis experiments. As shown in Supplemental Fig. 1, a 2.1 µM plasma AZM198 concentration diminished peritoneal MPO activity by more than 95%, arguing for adequate exposure to AZM198 in the HFD/AngII animals.

Plasma myeloperoxidase (MPO) protein concentrations were determined to investigate the effects of the lifestyle regiment and AZM198 treatment. As shown in Fig. 1C, plasma MPO levels were significantly elevated in the HFD/AngII group as compared to the control (LFD) group. This confirms the systemic inflammatory state under this condition. We like to note that this elevation was primarily driven by HFD and not by the low dose AngII treatment (Supplemental Fig. 2). Interestingly, MPO levels were normalized by AZM198 treatment to levels comparable to the control (LFD) group (Fig. 1C).

To confirm drug safety, a mouse group on control (LFD) diet received AZM198. Also in these animals, blood plasma MPO levels were significantly lower, but no side-effects were observed, confirming drug safety (Supplemental Table 3). Importantly, also no hematological differences, including neutrophil counts, were observed with the use of AZM198 (Supplemental Table 3).

Thus, not only MPO activity is affected by AZM198, but also MPO plasma protein levels. Whether this is a direct effect of AZM198 on MPO or an indirect effect, due to an altered inflammatory state, is currently not known. Together, these data show that HFD/AngII treatment resulted in elevated MPO levels and that AZM198 reduces MPO activity and plasma levels.

### AZM198 attenuated fat tissue accumulation

**Figure 2:**
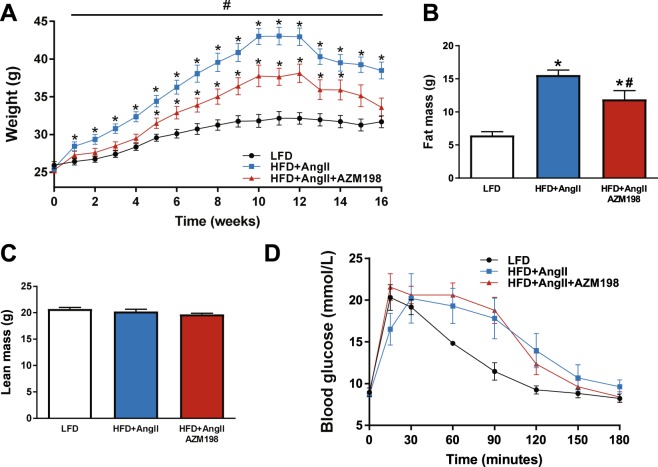
Bodyweight and glucose tolerance. The effects of myeloperoxidase (MPO) inhibition by AZM198 on bodyweight development, and glucose tolerance in the obesity/hypertension model induced by high fat diet (HFD) and angiotensin II (AngII) infusion. Control animals received low fat diet (LFD). (**A**) Bodyweight development pre- and post-pump implantation. Bodyweight is corrected for pump weight from week 13 and onwards. N = 11–12. (**B**) Quantification of total body fat mass from body composition analysis, corrected for pumps. (**C**) Quantification of total body lean mass from body composition analysis, corrected for pumps. (**D**) Serial blood glucose measurements in mice during intraperitoneal glucose tolerance testing (IPGGT). N = 6–7 for IPGTT. N = 11–12 for all other graphs. Bars and points on graph represent means. Error bars represent standard error of the mean. *P < 0.05 as compared to LFD. ^#^P < 0.05 as compared to HFD + AngII.

To investigate the effect of MPO inhibition on obesity, bodyweight changes of the mouse groups were determined weekly, starting at baseline. Already after 1 week of HFD, bodyweight of these mice was higher as compared to mice receiving LFD (Fig. 2A) and this was maintained throughout the experiment. Treatment with AZM198 did, however, attenuate bodyweight increase within 1 week of AZM198 treatment and this difference was sustained throughout the experiment. Analysis of body mass composition by using a minispec revealed that changes could be fully devoted to changes in fat mass accumulation without affecting lean mass, and fat mass accumulation was attenuated in the AZM198 treated group (Fig. 2B,C). An intra-peritoneal glucose tolerance test (IPGTT) at 16 weeks confirmed the pre-diabetic state of the HFD groups, but did not reveal any difference between the AZM198 treated and untreated groups (Fig. 2D). Thus, AZM198 attenuated bodyweight increase upon HFD by reducing fat tissue accumulation.

### Fat tissue inflammation and macrophage presence is reduced by AZM198

**Figure 3:**
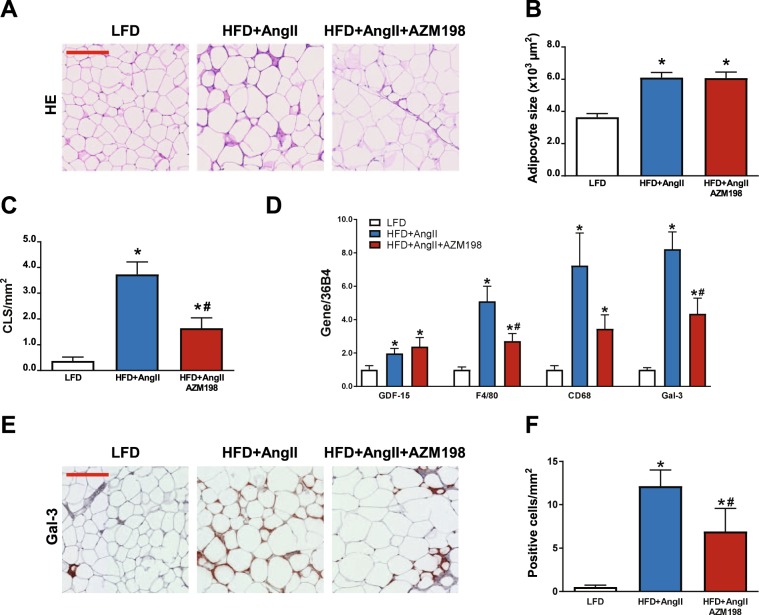
Inflammatory profile of visceral adipose tissue. The effects of myeloperoxidase (MPO) inhibition by AZM198 on visceral adipose tissue (VAT) inflammation in the obesity/hypertension model induced by high fat diet (HFD) and angiotensin II (AngII) infusion. Control animals received low fat diet (LFD). (**A**) Representative images of HE stained VAT sections, showing adipocyte size and crown-like structures (CLS). Red bar = 200 µm. (**B**) Quantification of adipocyte size from hematoxilin/eosin (HE) stained VAT sections. (**C**) Quantification of the number of inflammatory crown-like structures (CLS) from HE stained VAT sections as a measure for inflammatory cells. (**D**) Relative mRNA expression of growth differentiation factor 15 (GDF-15) and genes associated with macrophages, including F4/80, cluster of differentiation 68 (CD68) and galectin-3 (Gal-3). (**E**) Representative images of Gal-3 stained VAT sections, showing macrophages in VAT tissue. Red bar = 200 µm. (**F**) Quantification of macrophage presence from Gal-3 stained VAT. Gene expression values are corrected for 36B4 gene expression and presented as fold change. N = 8–11. Bars represent means. Error bars represent standard error of the mean. *P < 0.05 as compared to LFD. ^#^P < 0.05 as compared to HFD + AngII.

To further investigate the effects of MPO inhibition on adipose tissue, we performed gene expression analysis and histological stainings in VAT. HE staining in VAT sections showed that adipose cell size was similarly elevated in both AZM198 untreated and treated HFD groups (Fig. 3A,B). However, in the latter inflammatory crown–like structures were clearly less abundant, suggesting that AZM198 attenuates adipose tissue inflammation (Fig. 3A,C). The adipose stress factor growth differentiation factor-15 (GDF-15) was equally up-regulated in the AZM198 treated and untreated groups (Fig. 3D), but elevation of macrophage markers (F4/80, CD86, Gal-3) in adipose tissue of the AZM198 treated HFD/AngII group was significantly attenuated (Fig. 3D). To confirm diminished macrophage presence in adipose tissue in the AZM198 group, histological sections of VAT were stained with anti-Gal-3, a macrophage marker. Quantification showed that macrophage presence was clearly diminished in the AZM198 treated HFD/AngII group (Fig. 3E,F). Together this indicates that AZM198 can reduce HFD/AngII induced adipose tissue inflammation and adipose tissue macrophages counts.

### AZM198 attenuated liver degeneration

**Figure 4:**
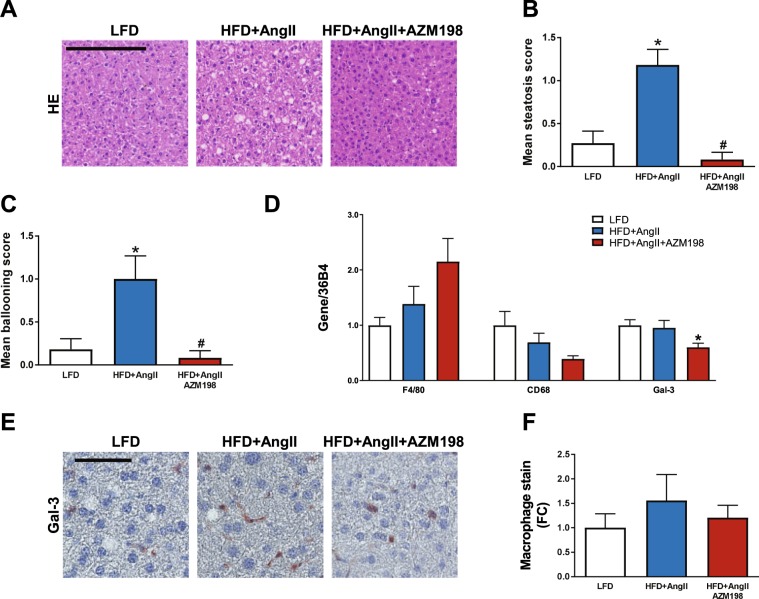
Liver degeneration and macrophages. The effects of myeloperoxidase (MPO) inhibition by AZM198 on liver degeneration and macrophage presence in the obesity/hypertension model induced by high fat diet (HFD) and angiotensin II (AngII) infusion. Control animals received low fat diet (LFD). (**A**) Representative images of the hematoxilin/eosin (HE) staining in liver sections, showing liver steatosis and ballooning. Black bar = 200 µm. (**B**) Quantification of the degree of steatosis from HE stained liver sections. (**C**) Quantification of the degree of ballooning from HE stained liver sections. (**D**) Relative mRNA expression in liver tissue of genes associated with macrophages, including F4/80, cluster of differentiation 68 (CD68) and galectin-3 (Gal-3). (**E**) Representative images of Gal-3 stained liver sections, showing macrophages in liver tissue. Black bar = 60 µm. (**F**) Quantification of macrophage presence from Gal-3 stained liver sections, represented as fold change. Gene expression values are corrected for 36B4 gene expression and presented as fold change. N = 8–12. Bars represent means. Error bars represent standard error of the mean. *P < 0.05 as compared to LFD. ^#^P < 0.05 as compared to HFD + AngII.

Besides adipose tissue, the liver is an important organ in fat metabolism and liver degeneration is a common symptom in obesity. Histological analysis of mouse liver tissue revealed strongly elevated liver steatosis and ballooning of hepatocytes in the HFD/AngII group (Fig. 4A–C). No liver fibrosis was observed in the HFD/AngII group (Supplemental Fig. 3A,B), indicative for early stage liver disease^36,37^. Interestingly, AZM198 treatment attenuated both liver steatosis and ballooning. To investigate whether macrophage presence was again a determining factor, macrophage gene expression was investigated. Only Gal-3 expression was significantly reduced in the AMZ198 group (Fig. 4D). This may suggest that whilst overall macrophage numbers did not change, the number of activated macrophages (marked by CD68) and M2 macrophages (marked by Gal-3) in liver were reduced in AZM198 treated mice. Histological analysis did not reveal clear differences in liver macrophages (Fig. 4E,F). Together, these data show that AZM198 was able to attenuate liver degeneration.

### Treatment with AZM198 had no effects on cardiac parameters

**Figure 5:**
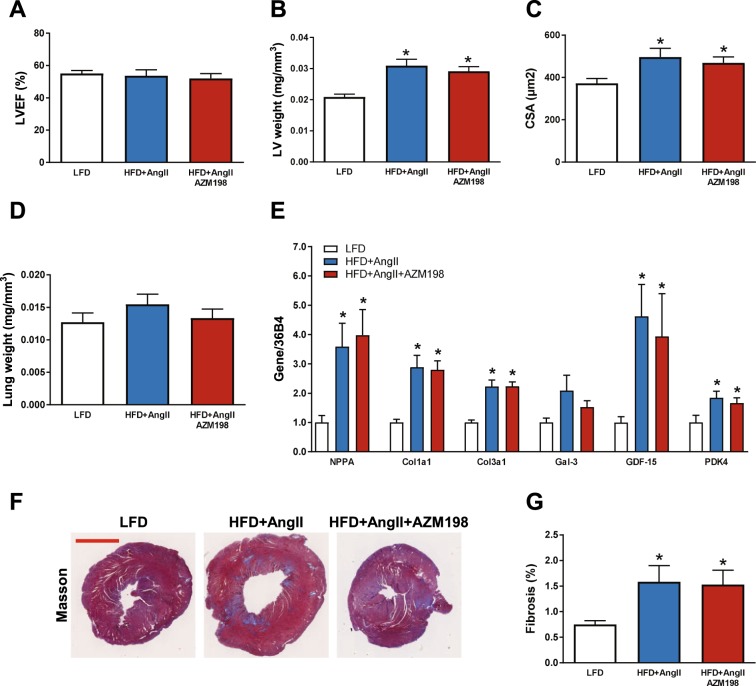
The effects of AZM198 on cardiac remodeling and function. The effects of myeloperoxidase (MPO) inhibition by AZM198 on measures of cardiac function and remodeling in the obesity/hypertension model induced by high fat diet (HFD) and angiotensin II (AngII) infusion. Control animals received low fat diet (LFD). (**A**) Quantification of left ventricular ejection fraction (LVEF) as determined by cardiac magnetic resonance imaging (CMR). (**B**) Left ventricular (LV) weight corrected by tibia length to the power of 3. (**C**) Quantification of LV cardiomyocyte cross sectional area (CSA) from WGA-FITC stained mid-ventricular sections. (**D**) Lung weight corrected by tibia length to the power of 3. (**E**) Relative mRNA expression of LV genes, including natriuretic peptide precursor type A (NPPA), alpha-1 type 1 collagen (Col1a1), alpha-3 type 1 collagen (Col3a1), galectin-3 (Gal-3), growth differentiation factor 15 (GDF-15) and pyruvate dehydrogenase kinase 4 (PDK4). (**F**) Representative images of Masson’s trichrome-stained mid-ventricular sections. Red bar = 2 mm. (**G**) Quantification of LV fibrosis from Masson’s trichrome-stained mid-ventricular sections. Gene expression values are corrected for 36B4 gene expression and presented as fold change. N = 10–12. Bars represent means. Error bars represent standard error of the mean. *P < 0.05 as compared to LFD.

As described before, the used obese/hypertensive mouse model generates a cardiac phenotype with HFpEF characteristics^38^. We therefore investigated multiple cardiac parameters to determine the effect of AZM198 on cardiac function in this mouse model. Hemodynamic measurements confirmed that LV end-systolic pressure (Pes) was increased in the HFD/AngII groups, confirming adequate AngII infusion (Table 1). Cardiac magnetic resonance imaging (CMR) measurements showed that LV ejection fraction (LVEF) was preserved (Fig. 5A and Table 1). Cardiac contraction (dP/dT_max_) and relaxation (dP/dT_min_) were impaired in the HFD/AngII groups, and AZM198 did not alter this (Table 1). Cardiac hypertrophy was present in the HFD/AngII mouse groups as determined by LV weight and cardiomyocyte size measurements (Fig. 5B,C). No significant changes in lung weight were observed, though the HFD/AngII group appeared to have slightly increased lung weight as compared to the LFD and AZM198 treated HFD/AngII groups (Fig. 5D). AZM198 had no effects on these parameters. Moreover, increased pathological cardiac gene expression (NPPA, encoding natriuretic peptide precursor type A) and increased expression of fibrotic genes (collagen genes: Alpha-1 type 1 collagen and alpha-3 type 1 collagen, Col1a1 and Col3a1), inflammatory genes (Gal-3 and GDF-15) and metabolic genes (Pyruvate dehydrogenase kinase 4, PDK4) were observed in the HFD/AngII mouse groups, but again AZM198 did not affect any of these parameters (Fig. 5E). LV fibrosis was also confirmed by Masson’s trichrome staining and showed a similar result for the HFD/AngII groups with and without AZM198 (Fig. 5F,G). Together, this indicates that in the obese/hypertensive mouse model AZM198 had no important cardioprotective effects.

**Table 1:** Hemodynamic measurements and cardiac dimensions in obese/hypertensive mice treated with AZM198.

	LFD	HFD + AngII	HFD + AngII AZM198
P-cathether			
Heart rate (bpm)	485 ± 21	460 ± 30	475 ± 29
LV Ped (mmHg)	10.5 ± 2.0	17.7 ± 1.8	18.7 ± 4.8
LV Pes (mmHg)	97.3 ± 2.3	114.1 ± 9.2*	118.7 ± 3.2*
LV dP/dt_max_ (1/s)	74.8 ± 4.7	59.0 ± 4.8*	64.1 ± 4.4
LV dP/dt_min_ (1/s)	−66.4 ± 6.4	−47.4 ± 4.1*	−47.2 ± 4.1
CMR			
LVEDV (µL)	49.9 ± 2.1	51.5 ± 2.5	55.9 ± 3.9
LVESV (µL)	22.7 ± 1.8	24.7 ± 3.1	27.6 ± 3.5
LVEF (%)	55.1 ± 1.8	53.7 ± 3.7	52.1 ± 2.9

Data are presented as means ± standard error of the mean. LFD = Low fat diet. HFD = High fat diet. AngII = Angiotensin II. AZM198 = Myeloperoxidase inhibitor. P-catheter = Pressure cathether. LV = Left ventricle. Ped = End-diastolic pressure. Pes = End-systolic pressure. dP/dt_max_ = Maximal LV contraction corrected by maximal LV pressure. dP/dt_min_ = Maximal LV relaxation corrected by maximal LV pressure. CMR = Cardiac magnetic resonance imaging. LVEDV = Left ventricular end-diastolic volume. LVESV = Left ventricular end-systolic volume. LVEF = Left ventricular ejection fraction. *P < 0.05 as compared to LFD.

## Discussion

In this study we investigated the effects of the novel myeloperoxidase (MPO) inhibitor AZM198 on obesity, liver damage and cardiac function in an obese and hypertensive mouse model, provoked by a high fat diet (HFD) combined with angiotensin II (AngII) infusion (HFD/AngII model). Our data show that MPO blood plasma levels indeed are increased in this model, indicative for a systemic inflammatory state. AZM198 could be detected in blood plasma at levels that provide approximately 95% MPO activity inhibition. AZM198 also normalized the MPO blood plasma protein levels in the HFD/AngII model to control levels. No adverse events were observed with AZM198 treatment. Interestingly, AZM198 reduced bodyweight increase by attenuating fat tissue inflammation. In particular, a reduction in inflammatory crown-like structures and macrophages in visceral adipose tissue (VAT) was observed. Moreover, steatosis and ballooning degeneration in the liver were reduced in the AZM198 treated HFD/AngII group. Potential AZM198 cardioprotective effects could, however, not be observed in this study.

Targeting cardiovascular risk factors, including obesity, is known to improve cardiovascular prognosis^39^. The association between obesity, inflammation and cardiovascular diseases suggests anti-inflammatory therapies could be an interesting therapeutic approach in cardiovascular risk management^9,11–16,39^. MPO^−/−^ knockout mice show normal development and have no apparent phenotype under standard conditions^40^, but show reduced bodyweight gain and a reduction in crown-like structures and inflammatory markers in VAT after HFD feeding^29^. In concordance, we observed that MPO inhibition through AZM198 treatment was associated with positive effects on obesity and adipose tissue inflammation. Moreover, obesity and non-alcoholic steatohepatitis (NASH) are closely linked. With increased availability of fatty acids, which are derived from adipose tissue, development of liver steatosis is promoted^41^. In a previous study, NASH induced liver damage was reduced in MPO^−/−^ knockout mice^27^. Also in our study mice showed a NASH-like phenotype, and with AZM198 treatment liver damage was prevented as shown by a normalization of steatosis and ballooning scores. Thus, our results are therefore in line with these previous studies with genetically engineered MPO knockout mice.

Inflammatory cells present in adipose tissue, including macrophages, are important producers of inflammatory cytokines and are therewith an important driver of the pro-inflammatory state associated with obesity^42^. This pro-inflammatory state is also suggested to play an important role in the development of cardiovascular diseases, including HFpEF^9,11–16^. In this study, VAT macrophage expansion was markedly reduced by AZM198 treatment, and this was true for F4/80 (mature macrophage marker), CD68 (activated macrophage marker) and Gal-3 (M2-macrophage marker) sub-types. In liver on the other hand, the total number of F4/80 macrophages seemed unaffected by AZM198, but there was a reduction in the number of activated CD68 macrophages and M2 macrophages. These effects of AZM198 on macrophages in VAT and liver could possibly explain the attenuating effects of AZM198 on obesity and liver damage. The positive effects of AZM198 on the liver could also be indirectly mediated via the positive effects on adipose tissue, and this requires further investigations. All together these results show that MPO inhibition by AZM198 could be an interesting new pharmacological approach to diminish adipose tissue inflammation and liver degeneration.

Obesity and hypertension are important risk factors for cardiovascular diseases, including HFpEF^9,11–16^. Studies in MPO knockout mice have shown that MPO is involved in cardiac remodeling. In post myocardial infarcted MPO^−/−^ knockout mice, thinning of the ventricular walls was reduced and ventricular dilatation was attenuated^32,43^. Though an inflammatory state was induced in the model used in our study, the cardiac phenotype was not attenuated by AZM198 treatment. Since the degree of cardiac inflammation and remodeling in the used obese/hypertensive mouse model is small as compared to previous reported cardiac remodeling in the infarction and other severe HF models, it is possible that the effects of AZM198 on cardiac remodeling were too subtle to be observed. Moreover, MPO protein levels were about 3.8 ± 0.6 and 4.3 ± 1.1 times higher in liver and VAT as compared to the heart, which could explain why the main effects of AZM198 were observed in liver and VAT.

AZM198 is a relatively novel MPO inhibitory drug belonging to the class of 2-thioxanthines, and this class of drugs has shown good specificity towards MPO^34,35,44^. This drug shows high specificity towards MPO, as compared to other peroxidases, like the structurally related thyroid peroxidase (TPO)^34^. A previous study, using a highly specialized MRI/microPET method and a specific MPO-activated tracer, described up to 80% reduction of MPO activity by AZM198 in aortic plaques^34^. Moreover, other studies showed that a 2 uM plasma level of AZM198 resulted in in almost complete MPO inhibition, which was confirmed by our study^34,35^. We like to note that predominantly extracellular MPO is targeted at these AZM198 plasma levels and neutrophil intragranular MPO inhibition has been shown to require higher AZM198 levels^35^. Our study is one of the first describing AZM198 treatment in disease models. Thus far, no side effects at these therapeutic levels of the drug have been reported, and also in our study no harmful side effects, including hematological side effects, were observed.

In studies performed in HF populations, MPO blood plasma levels were associated with HF severity and prognosis^23–25^. Recently, cardiac specificity of several novel HF biomarkers has been questioned, whilst cardiac specificity is important to directly correlate the level of a circulating marker to the degree of cardiac remodeling^38,45^. In our mouse study the elevated plasma MPO levels were primarily observed in association to HFD, whereas the cardiac phenotype was predominantly dependent on the low dose AngII infusion. This is different from previously reported high dose AngII infusion studies in which MPO plasma levels were elevated^30^. Therefore, the AngII induced cardiac remodeling in our mouse model is less MPO driven and this may also explain the lack of improvement of cardiac function by AZM198 treatment. These data also indicate that MPO levels are not indicative for cardiac function and remodeling and may limit its role as a heart failure biomarker. On longer term, sustained obesity might very well translate into long term stress to the heart and incident heart failure, so that our results clearly do not preclude a beneficial effect on the long term. The lag time between the onset of obesity and incident HFpEF in obese individuals is typically 1–2 decades. In this relatively short term mouse model we could not study these important long term effects.

Several limitations of this study should be mentioned. First, whilst in humans, development of obesity and hypertension is a slow process that takes decades, the induction of the model in this study was relatively fast. When studied in a more prolonged time window or in a more severe model, cardiac protective effects of AZM198 might be observed and also effects on liver fibrosis could be studied. Second, only two cardiovascular risk factors were included in this study, namely obesity and hypertension. AZM198 may show protective effects on the pro-inflammatory states induced by other cardiovascular risk factors, including age, kidney disease and chronic obstructive pulmonary disease (COPD) amongst others^4,16^. Whereas in this study positive AZM198 effects on liver and VAT were observed, cardiac remodeling and function were not altered. This does not exclude the possibility that cardiac protective effects of AZM198 may be present in other heart failure animal models, such as described for PF-1355 in post myocardial infarct remodeling^33^, or at different dosage regimes and this will require further investigations. Finally, because direct MPO measurements for plasma or tissue are not available, we used indirect methods to determine MPO inhibition levels. Although this is a limitation, our observations were in line with other studies^34,35^. This study was designed to investigate the effects of AZM198 on multiple tissues with a cardiovascular scope. The observed protective effects on adipose and liver tissues now set the stage for more focused studies to delineate the molecular mechanisms in these tissues in association to MPO-inhibition by AZM198.

Altogether, we have shown that plasma MPO levels are elevated in obese/hypertensive mouse model and that AZM198 was able to reduce blood plasma MPO levels. Fat tissue accumulation, adipose inflammation and liver degeneration were effectively reduced by AZM198. We postulate that by attenuating the severity of these important cardiovascular precipitating factors, prolonged pharmacological inhibition of MPO by AZM198 may have value in cardiovascular risk reduction and cardiovascular prevention.

## Methods

### Animals

The animal experimental protocol was approved by the Animal Ethical Committee of the University of Groningen (permit numbers: AVD105002016487 and IVD16487-03-01). All procedures were performed conform the existing guidelines for the care and use of laboratory animals. Male C57BL/6 J mice (Envigo, the Netherlands) were housed on a 12/12 hours day/night cycle in a controlled environment and ad libitum access to water and chow. Mice were randomized based on bodyweight at an age of 8 weeks to create the experimental groups. Mice with abnormal bodyweight (>2 SD) or showing abnormal behavior were excluded. Prior to surgery, mice received a subcutaneous dose of carprofen (5.0 mg/kg) to reduce post-operative wound pain. Functional measurements, including cardiac magnetic resonance imaging (CMR) and hemodynamic measurements, and all molecular and histological analysis were performed in a blinded fashion.

### Mouse experimental protocol

Mice received a high fat diet (HFD) (60 kcal% fat, D12492, Research diets, USA), or a control low fat diet (LFD) (10 kcal% fat, D12450J, Research Diets, USA). The AZM198 treatment group received 0.16% AZM198 in the diet. As a control, no AZM198 was added. To exclude differences in food intake between experimental groups, food intake was measured for a selection of animals of each group for a period of 12 consecutive days.

After 12 weeks of diet intervention, HFD mice were randomized to receive 4 weeks of either human angiotensin II (AngII) (1 mg/kg/day dissolved in saline, Bachem, Switzerland) or saline infusion using osmotic minipumps (Alzet 1004, Durect corporation, USA). Pumps were combined with polyetheretherketone tubing (Alzet PEEK tubing 0002612, Durect corporation, USA) to allow CMR measurements. Pump placement was performed as described before^46^. In brief, mice were anesthetized with 2% isoflurane/oxygen via inhalation and were placed in the prone position on a heating pad to maintain body temperature. A subcutaneous pocket was created in the right flank for pump insertion. After placing the pump, the skin was closed using a 5-0 silk suture.

Prior to sacrifice, bodyweight was determined and body mass composition analysis was performed to determine body fat content. Both bodyweight and body mass composition were corrected for the implanted mini-pumps. An intraperitoneal glucose tolerance test was performed to investigate diabetic status. Also, cardiac function was determined using CMR measurements. Mice were sacrificed at 16 weeks. A timeline of the experiment is depicted in Fig. 1A.

### *In vivo* AZM198 potency analysis

The potency of AZM198 on tissue MPO activity was determined in separate peritonitis experiments at AstraZeneca, Sweden (ethical permit number 74-2011). Female BALB/c mice (Charles River Labs, Germany) were primed with an intraperitoneal injection of thioglycollate broth, followed by an intraperitoneal challenge with zymosan (Sigma-Aldrich, USA) 24 hours later. After an additional 2 hours, the mice were perorally gavaged with a single dose of 0.5, 1.5, 5, 10, 50 or 250 µmol/kg AZM198, or the vehicle 1% hydroxypropylmethylcellulose. Two hours after gavage, the peritoneal cavity was rinsed with phosphate-buffered saline. The cells were spun down and the MPO activity in the supernatant was determined by chemiluminescence after reactivation with H_2_O_2_ in the presence of luminol. Data are expressed as percent of the mean response of the vehicle-treated controls and represent two independent experiments.

### Body mass composition measurements

Body mass composition, including total body fat content and total body lean content, was determined using a minispec LF90II body composition analyzer (Bruker Optics, USA).

### Glucose tolerance test

Mice were fasted for 6 hours and, subsequently, injected intraperitoneally with a sterile 2 g/kg glucose solution (50% glucose in 0,9% NaCl, Merck, Germany) using sterile insulin syringes (U-100 Insulin, Terumo, Japan). Blood was drawn from the tail vein immediately before and 15, 30, 60, 120, 150 and 180 minutes after glucose injection. Blood glucose levels were determined using an Accu-Check Aviva glucose analyzer (Roche Diagnostics, Germany).

### CMR measurements

CMR measurements were performed just before sacrifice using a vertical 9.4-T, 89 mm bore size magnet equipped with 1500 mT/m gradients combined with a 400 MR system (Bruker Biospin, Germany). CMR measurements were performed as described before^47^. Mice were anesthetized with 2% isoflurane/oxygen. Respiration and cardiac electrophysiology, including heart rate, were monitored using an electrocardiography (ECG) trigger unit (RAPID biomedical, GmBH, Germany). By slightly adjusting the dosage of isoflurane, heart rate and respiration rate were maintained between 400–600 and 20–60, respectively. Depending on the size of the heart, 7–9 two dimensional images were recorded to cover the whole left ventricle (LV). CMR acquisition and reconstruction were performed using ParaVision and IntraGate software (Bruker Biospin, GmBH, Germany). Using semi-automatic contour detection software (QMass, version MR 6.1.5, Medis Medical Imaging systems, the Netherlands), left ventricular end-diastolic volume (LVEDV) and left ventricular end-systolic volume (LVESV) were determined, and left ventricular ejection fraction (LVEF) was calculated.

### Hemodynamic measurements and sacrifice

Euthanasia of animals was performed under anesthesia, but prior to sacrifice hemodynamic measurements were performed. Mice were anesthetized with 2% isoflurane/oxygen. A catheter (Scisence catheter 1.2 F, Transonics, USA) was inserted via the right carotid artery. Parameters of cardiac function were recorded, including LV pressures, LV dP/dt_max_ (a measure for maximal LV contraction capacity), LV dP/dt_min_ (a measure for maximal LV relaxation capacity) and heart rate. After catheter removal, blood was collected and transferred into ethylenediaminetetraacetic acid (EDTA) tubes. One portion of blood designated for complete blood count was stored in EDTA tubes at 4 °C until analysis. A second portion of blood was centrifuged immediately after blood collection at 1500 g for 10 min and blood plasma was collected. To remove remaining red blood cells, organs and tissues were flushed with saline. Next, organs and tissues of interest were collected. LV weight and lung weight were determined. An LV mid-slice of each heart, a sample of visceral adipose tissue (VAT) and a piece of liver were fixed in formalin and processed for histology. Blood plasma, organs and tissues were frozen in liquid nitrogen and stored at −80 °C. Tibias were collected and measured. Organ weights were corrected for tibia length to the power of 3^48^.

### Complete blood counts

Complete blood count was determined on the day of sacrifice using a sysmex hematology analyzer (Symex XN-10, Sysmex Corporation, Japan). Before performing complete blood counts, blood was diluted 5 times with 0,9% NaCl.

### Plasma levels of MPO

Blood plasma MPO levels were determined in 20 times diluted plasma samples using MPO enzyme-linked immunosorbent assay (ELISA) kits (Hycult biotech, the Netherlands).

### Plasma levels of AZM198

Plasma levels of AZM198 were determined in 10 µL EDTA plasma using protein precipitation with acetonitrile followed by ultra-performance liquid chromatography (UPLC) and tandem mass spectrometry (UPLC-MS/MS). By addition of 180 µL of acetonitrile containing internal standard, followed by mixing and centrifugation, precipitation was obtained. Supernatant was diluted 1:1 with 0.2% formic acid. Subsequently, this mixture was injected on an ACQUITY UPLC HSS T3 1,8 µm 2.1 × 50 mm column, followed by eluting using a gradient mobile-phase profile (mobile phase A: 0.2% formic acid in water, mobile phase B: 0.2% formic acid in acetonitrile). Analytes were detected by multiple reaction monitoring (MRM). The lower limit of quantification (LLOQ) was 8 nmol/L.

### Histological assessment of VAT, liver and LV

Slices of 4 µm thickness were cut from formalin-fixed and paraffin-embedded VAT, liver tissue samples, and LV mid-transverse sections.

To visualize crown-like structures and adipocyte cell size in VAT, and steatosis and ballooning in liver tissue, deparaffinized sections were stained with hematoxilin (Mayers Hematoxilin, MHS32-1L, Sigma-Aldrich, USA) and eosin (Epsom U solution, HT110232-1L, Sigma-Aldrich, USA) and mounted by DPX mounting medium (Sigma-Aldrich, USA) followed by automatically imaging of whole stained sections using a Nanozoomer 2.0 HT. (Hamamatsu, Japan). Quantifying the amount of crown-like structures in sections was performed by counting the number of crown-like structures in 5 randomly picked areas, each with a size of 0.75 mm^2^. Adipocyte cell size was determined by counting the number of adipocytes in 5 randomly picked areas, each with a size of 0.15 mm^2^. Next, the mean adipocyte size was calculated by dividing the combined area size by the total number of adipocytes. Quantifying the degree of steatosis and ballooning in liver sections was performed using a histological scoring system for NASH, as described elsewhere^49^. In short, degree of steatosis and ballooning was scored on a scale of 0–3 and 0–2, respectively.

As a measure of macrophage presence, a galectin-3 (Gal-3) staining was performed on deparaffinized VAT and liver sections. Antigen retrieval was performed using 10 mM citrate buffer (Citric acid monohydrate, 100241, Merck, Germany) with a pH of 6.0. After blocking endogenous peroxidases with H_2_O_2_, sections were incubated with rat-anti-Mac2 (CL8942AP, Cedarlane, Canada) diluted 1:1500 in phosphate buffered saline (PBS) with 1% bovine serum albumin (BSA, 11930.03, Serva, Germany) for 1 hour, rabbit-anti rat IgG/HRP diluted 1:100 in PBS with 1% BSA for 45 min and AEC substrate-chromogen (K3464, Agilent, USA) for 8 min, followed by hematoxilin counterstaining (Mayers Hematoxilin, MHS32-1L, Sigma-Aldrich, USA). Sections were mounted using faramount mounting medium (S3025, Agilent, USA). Whole stained sections were automatically imaged using a Nanozoomer 2.0 HT. (Hamamatsu, Japan). In VAT, the number Gal-3 stained adipocyte cells was counted in 5 randomly picked areas, each with a size of 0.75 mm^2^. In liver, the fraction of positive stained pixels (AEC, red colour) of the entire section was determined using ImageScope software (ScanScope, Aperial Technologies, USA).

For histological assessment of liver fibrosis and cardiac fibrosis, deparaffinized liver and LV sections were stained with Picro Sirius Red Staining for collagen detection or Masson’s trichrome for collagen detection, respectively, followed by automatically imaging of whole stained sections using a Nanozoomer 2.0 HT (Hamamatsu, Japan). Quantification of fibrosis fraction as a percentage of the entire section was performed from a 20 fold magnification (ScanScope, Aperial Technologies, USA).

Cardiomyocyte size was determined on fluorescein-isothiocyanate (FITC) labeled wheat germ agglutinin (WGA, Sigma-Aldrich, USA) stained deparaffinized LV sections mounted in DAPI mounting medium (4’,6-diamidino-2-phenylinodole, Vector laboratories, USA), in order to visualize the nucleus. Five randomly selected fields from the WGA-stained LV sections were imaged using a Leica DMI6000B inverted fluorescent microscope (Leica Microsystems B.V., The Netherlands) and the cross sectional area (CSA) of approximately 40 cardiomyocytes per mouse heart was measured using Image J software (NIH, USA).

## RT-qPCR

Total RNA was extracted from powdered liver and LV tissue using Trizol reagent (Invitrogen, Thermo Fisher Scientific, USA) and from VAT using RNeasy lipid tissue mini kits (Qiagen, the Netherlands). RNA concentration of each sample was determined by spectrophotometry (NanoDrop 2000, Thermo Scientific, the Netherlands). cDNA was synthesized using QuantiTect Reverse Transcriptional kits (Qiagen, the Netherlands). In total, 7.5 ng cDNA was used to determine relative gene expression by real-time quantitative polymerase chain reaction (RT-qPCR) using the Bio-Rad CFx384 real time system (Bio-Rad, the Netherlands) and ABsolute SYBR Green mix (Thermo Scientific, the Netherlands). Gene expressions were corrected for ribosomal protein, large, P0 (36B4) reference gene expression and are presented as relative expression to the control group. Oligonucleotide pairs used for RT-qPCR are presented in Supplemental Table 1.

### Statistical analysis

Data are presented as means ± standard errors of the mean (SEM) for continuous variables and as absolute number with percentages for categorical variables. Normality of data and homogeneity of variance was tested using the Shapiro-Wilk test for normality and the Levene’s test for homogeneity of variances, respectively. Normally distributed data was analyzed with the independent-samples T-test for 2 group comparisons, or with one-way analysis of variance (ANOVA) followed by Tuckey’s post-hoc correction for multi-group comparisons. For non-normally distributed data or data that lacked homogeneity of variances, non-parametrical tests were used. Differences between two groups were analyzed using Mann-Whitney U tests. Multi-group comparisons were analyzed starting with Kruskal-Wallis tests and, subsequently, Mann-Whitney U tests. A P-value < 0.05 was considered significant. Statistical analysis was performed using SPSS software (IBM SPSS statistics, version 23, IBM, USA).

### Ethics approval

The animal experimental protocol of the AZM198 experiment in the HFD/ANGII model was approved by the Animal Ethical Committee of the University of Groningen (permit numbers: AVD105002016487 and IVD16487-03-01). The potency of AZM198 on tissue MPO activity was determined in peritonitis experiments at AstraZeneca, Sweden (ethical permit number 74-2011).

## Supplementary information

Supplementary Information

## Data Availability

The datasets used and/or analyzed during the current study are available from the corresponding author on reasonable request.
