# Supplementary material for: Pharmacological myeloperoxidase (MPO) inhibition in an obese/hypertensive mouse model attenuates obesity and liver damage, but not cardiac remodeling
Source: Sci Rep. 2019 Dec 10;9:18765. doi: 10.1038/s41598-019-55263-y (PMC6904581; doi:10.1038/s41598-019-55263-y)
Supplement: Supplementary file 1 — Supplementary Information [file 41598_2019_55263_MOESM1_ESM.pdf]

Supplementary information

Pharmacological myeloperoxidase (MPO) inhibition in an obese/hypertensive mouse model attenuates obesity and liver damage, but not cardiac remodeling

Arnold Piek[1], Debby P. Y. Koonen[2], Elisabeth-Maria Schouten[1], Eva L. Lindtstedt[3], Eric Michaëlsson[3], Rudolf A. de Boer[1] and Herman H.W. Silljé[1]

[1] Department of Cardiology, University Medical Center Groningen, University of Groningen, The Netherlands

[2] Department of Pediatrics, Section Molecular Genetics, University Medical Center Groningen, University of Groningen, the Netherlands

[3] Early Cardiovascular, Renal and Metabolism, R&D BioPharmaceuticals, AstraZeneca, Gothenburg, Sweden

Supplemental tables

Supplemental table 1. Oligonucleotide sequences used for qPCR

Gene	5' - 3' forward	5' - 3' reverse
CD68	AAAGGTAAAGCTAAAGTGGGGC	GGTAGACTGTACTCGGGCTCT
Col1a1	CTTCACCTACAGCACCCTTGTG	CTTGGTGGTTTTGTATTCGATGAC
Col3a1	ACGTAAGCACTGGTGGACAG	CAGGAGGGCCATAGCTGAAC
F4/80	TTTCCTCFCCTGCTTCTTC	CCCCGTCTCTGTATTCAACC
Gal-3	CCCGCTTCAATGAGAACAAC	ACCGCAACCTTGAAGTGGTC
GDF-15	TGACCCAGCTGTCCGGATAC	GTGCACGCGGTAGGCTTC
NPPA	ATGGGCTCCTTCTCCATCAC	TCTACCGGCATCTTCTCCTC
PDK4	GCATTTCTACTCGGATGCTCATG	CCAATGTGGCTTGGGTTTCC
36B4	AAGCGCGTCCTGGCATTGTC	GCAGCCGCAAATGCAGATGG

Supplemental table 2. Food intake measurements of mice included in the obesity/hypertension experiment.

	Placebo	AZM198	P-value
**LFD intake (gram)**			
Interval 1 (4 days)	12.3 ± 6.1	12.0 ± 0.5	0.885
Interval 2 (4 days)	12.9 ± 0.5	12.0 ± 0.2	0.146
Interval 3 (4 days)	12.2 ± 0.5	11.7 ± 0.3	0.564
Total	37.4 ± 1.4	35.7 ± 0.3	0.564
**HFD intake (gram)**			
Interval 1 (4 days)	11.3 ± 0.5	10.5 ± 0.5	0.139
Interval 2 (4 days)	10.3 ± 0.4	10.2 ± 0.7	0.309
Interval 3 (4 days)	11.5 ± 0.5	11.7 ± 1.2	0.663
Total	33.1 ± 1.3	32.3 ± 1.8	0.564

Data are presented as means ± standard error of the mean. AZM198=Myeloperoxidase inhibitor. LFD=Low fat diet. HFD=High fat diet. N=4. No statistical differences were observed.

Supplemental table 3. Safety of AZM198

Variable	LFD	LFD+AZM198	P-value
**Blood plasma levels**			
AZM198 (µM)	0.0 ± 0.0	2.1 ± 0.4	0.000*
MPO (ng/ml)	325.8 ± 33.6	226.5 ± 17.4	0.033*
**Survival**			
Premature deaths, n (%)	0 (0%)	0 (0%)	1.000
**Full blood analysis**			
Hb (mmol/L)	8.3 ± 0.2	8.1 ± 0.1	0.400
HCT (L/L)	0.43 ± 0.01	0.42 ± 0.01	0.423
RBC (10^12/L)	9.4 ± 0.2	9.4 ± 0.2	0.833
WBC (10^9/L)	3.9 ± 0.7	3.2 ± 0.5	0.453
Lymphocytes (10^9/L)	3.5 ± 0.6	2.9 ± 0.5	0.496
Neutrophils (10^9/L)	0.38 ± 0.09	0.24 ± 0.4	0.233
**Body and organ weight**			
Bodyweight (g)	31.7 ± 0.8	30.7 ± 0.7	0.388
LV weight (mg/ TL^3)	0.02 ± 0.00	0.02 ± 0.00	0.796
**CMR**			
LVEDV (µl)	49.9 ± 2.1	50.9 ± 2.6	0.870
LVESV (µl)	22.7 ± 1.8	22.7 ± 2.1	0.974
LVEF (%)	55.1 ± 1.8	56.2 ± 1.8	0.577
**P-cathether**			
Heart rate (bpm)	485 ± 21	471 ± 14	0.496
LV Ped (mmHg)	10.5 ± 2.0	13.9 ± 1.7	0.226
LV Pes (mmHg)	97.3 ± 2.3	101.9 ± 4.0	0.290
LV dP/dt_{max} (1/s)	74.8 ± 4.7	63.8 ± 3.1	0.096
LV dP/dt_{min} (1/s)	-66.4 ± 6.4	-57.6 ± 5.2	0.226
**LV histology**			
Cardiomyocyte CSA (μm^2)	372.5 ± 22.5	393.1 ± 19.2	0.492
Fibrosis (%)	0.8 ± 0.1	0.9 ± 0.1	0.490
**Fat tissue analysis**			
Fat mass (g)	6.4 ± 0.6	5.9 ± 0.8	0.412
VAT Adipocyte size ($x10^3$ μm^2)	3.6 ± 0.2	3.6 ± 0.3	0.983
VAT CLS/mm^2	0.4 ± 0.2	0.4 ± 0.2	0.890
**Liver tissue analysis**			
Mean steatosis score	0.3 ± 0.1	0.1 ± 0.1	0.326
Mean ballooning score	0.2 ± 0.1	0.0 ± 0.0	0.167

Data are presented as means ± standard error of the mean for continuous variables and as N (%) for categorical variables. LFD=Low fat diet. AZM198=Myeloperoxidase inhibitor. MPO=Myeloperoxidase. Hb=Hemoglobin. HCT=Hematocrit. RBC=Red blood cells. WBC=White blood cells. LV=Left ventricle. CMR=Cardiac magnetic resonance imaging. LVEDV=Left ventricular end-diastolic volume. LVESV=Left ventricular end-systolic volume. LVEF=Left ventricular ejection fraction. P-catheter=Pressure catheter. Ped=End-diastolic pressure. Pes=End-systolic pressure. dP/dt_{max}=Maximal LV contraction corrected by maximal LV pressure. dP/dt_{min}=Maximal LV relaxation corrected by maximal LV pressure. CSA=Cross sectional area. VAT=Visceral adipose tissue. CLS=Crown like structures. N=8-11. *P<0.05 as compared to LFD.

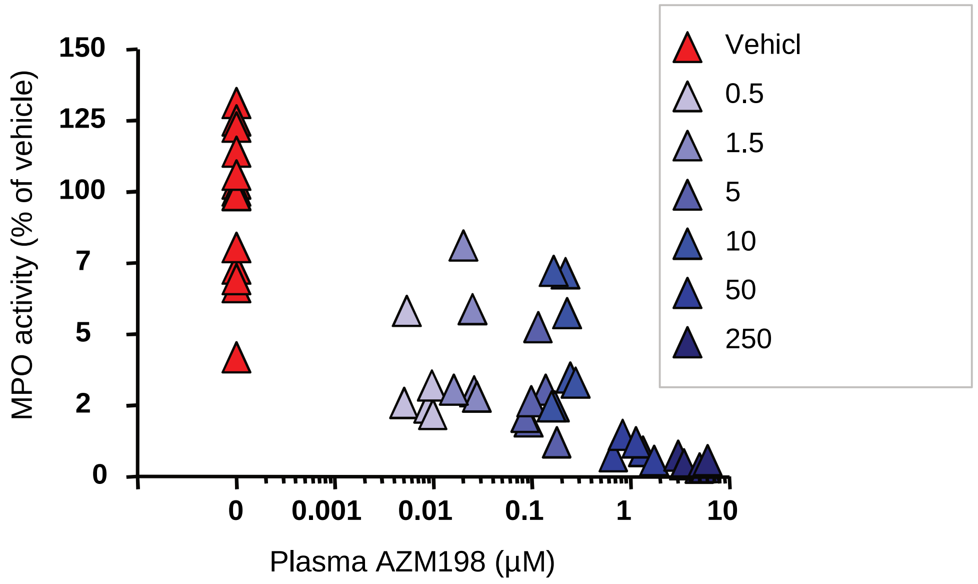

Supplemental figure 1. Effect of plasma AZM198 concentration on MPO activity in zymosan-induced peritonitis

Myeloperoxidase (MPO) activity was determined in peritoneal fluid after thiglyconate and zymosan induced peritonitis. AZM198 was administered 2 hours after zymosan treatment and peritoneal fluid was collected 2 hours later. AZM198 levels in blood plasma and MPO activity in peritoneal fluid were subsequently determined.

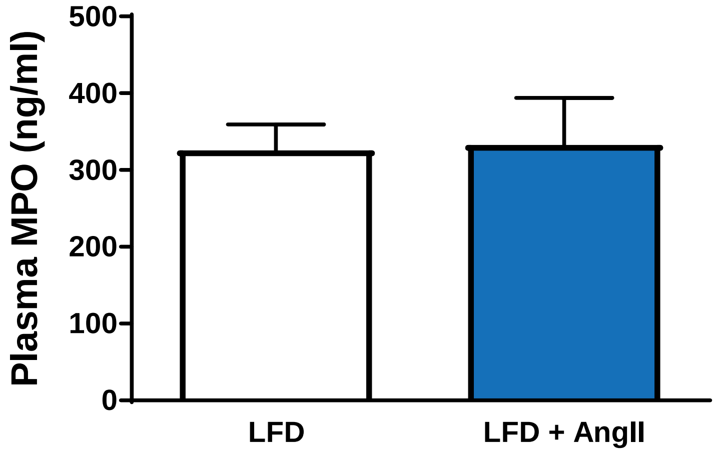

Supplemental figure 2. MPO plasma levels

Blood plasma myeloperoxidase (MPO) levels of animals receiving low fat diet (LFD) combined with either saline or angiotensin II (AngII) infusion. N=11-13. Bars represent means. Error bars represent standard error of the mean. No statistical differences were observed.

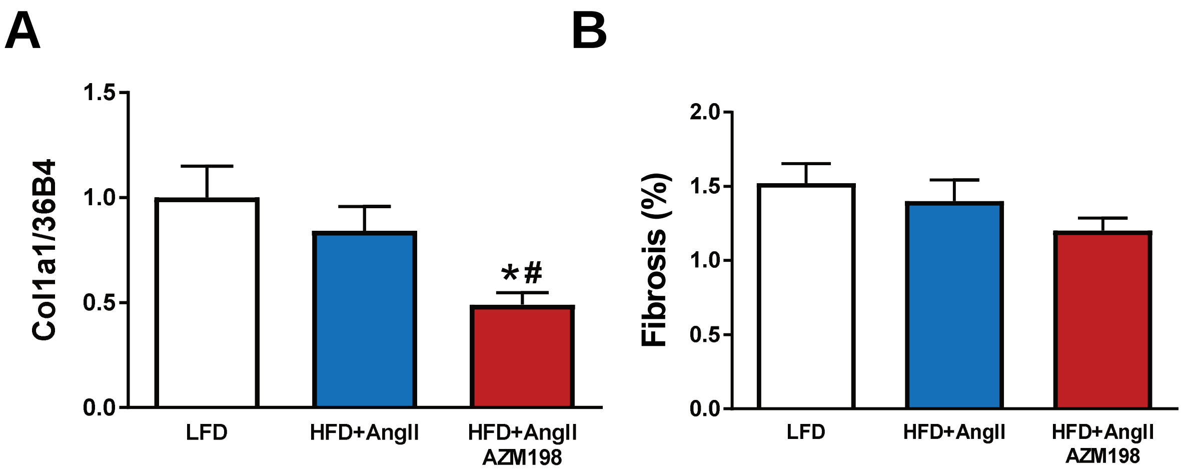

Supplemental figure 3. Liver fibrosis

Liver fibrosis in the obesity/hypertension model induced by high fat diet (HFD) and angiotensin II (AngII) infusion. The intervention group was treated with myeloperoxidase (MPO) inhibitor (AZM198). Control animals received low fat diet (LFD). (A) Relative mRNA expression of alpha-1 type 1 collagen (Col1a1) in liver. (B) Quantification of liver fibrosis from Picro Sirius Red stained liver sections. Gene expression values are corrected for 36B4 gene expression and presented as fold change. N=8-12. Bars represent means. Error bars represent standard error of the mean. *P<0.05 as compared to LFD. #P<0.05 as compared to HFD/AngII.
